# Gamma-Glutamyl Transpeptidase to Platelet Ratio: A New Inflammatory Marker Associated with Outcomes after Cardiac Arrest

**DOI:** 10.1155/2021/5537966

**Published:** 2021-08-13

**Authors:** Yipin Zhao, Zebin Lin, Yingying Ji, Huawei Wang, Li Xiao, Qingwei Chen, Zhiqin Wu

**Affiliations:** ^1^Department of General Medicine, The Second Affiliated Hospital of Chongqing Medical University, Chongqing, China; ^2^Department of Intensive Care Unit, The Second Affiliated Hospital of Zhengzhou University, Zhengzhou 450014, China

## Abstract

**Introduction:**

In recent years, gamma-glutamyl transpeptidase to platelet ratio (GPR) has been proposed as a new inflammatory marker. We aimed to evaluate the association between GPR and outcomes after cardiac arrest (CA).

**Methods:**

A total of 354 consecutive patients with CA were included in this retrospective study. Patients were divided into three groups according to tertiles of GPR (low, *n* = 119; middle, *n* = 117; and high, *n* = 118). To determine the relationship between GPR and prognosis, a logistic regression analysis was performed. The ability of GPR to predict the outcomes was evaluated by receiver operating characteristic (ROC) curve analysis. Two prediction models were established, and the likelihood ratio test (LRT) and the Akaike Information Criterion (AIC) were utilized for model comparison.

**Results:**

Among the 354 patients (age 62 [52, 74], 254/354 male) who were finally included in the analysis, those in the high GPR group had poor outcomes. Multivariate logistic regression analysis revealed that GPR was independently associated with the three outcomes, for ICU mortality (odds ratios (OR) = 1.738, 95% confidence interval (CI): 1.221-2.474, *P* = 0.002), hospital mortality (OR = 1.676[1.164 − 2.413], *P* = 0.005), and unfavorable neurologic outcomes (OR = 1.623[1.121 − 2.351], *P* = 0.010). The area under the ROC curve was 0.611 (95% Cl: 0.558-0.662) for ICU mortality, 0.600 (95% CI: 0.547-0.651) for hospital mortality, and 0.602 (95% CI: 0.549-0.653) for unfavorable neurologic outcomes. Further, the LRT analysis showed that compared with the model without GPR, the GPR-combined model had a higher likelihood ratio *χ*^2^ score and smaller AIC.

**Conclusion:**

GPR, as an inflammatory indicator, was independently associated with outcomes after CA. GPR is helpful in estimating the clinical outcomes of patients with CA.

## 1. Introduction

Cardiac arrest (CA) is one of the leading causes of death worldwide. In the United States, there are about 350,000 cases of out-of-hospital cardiac arrest (OHCA) and 200,000 cases of in-hospital cardiac arrest (IHCA) annually [1, 2], with similarly high numbers in Europe. [3]. Despite advances in treatment concepts and strategies, the prognosis of patients with CA remains poor. The overall survival rates to hospital discharge after OHCA and IHCA were both very low, approximately 12% and 24%, respectively [4–6]. A high proportion of surviving patients present with neurological deficits, often accompanied by disability and high morbidity due to CA-induced hypoxic-anoxic ischemic brain injury [7, 8]. Each CA patient has different clinical conditions and needs different treatment, and multiple clinical parameters influence the clinical outcome of CA [9]. In clinical practice, it is of great significance to find useful biomarkers as simple prognostic indicators to accurately identify the individuals with poor prognosis in the early stage of CA, formulate personalized treatment strategies, and improve the success rate of rescue treatment. The American Heart Association, the European Resuscitation Council, and the European Society of Intensive Care Medicine recommend using a combination of multiple predictors such as clinical examination, blood parameters, electrophysiological measurements, and imaging findings to assess prognosis [1, 10, 11]. However, current guidelines and research are more focused on neurological prognosis.

Cardiac arrest is one of the causes of systemic inflammatory response syndrome (SIRS) [12]. SIRS is activated immediately during cardiac arrest and after the return of spontaneous circulation (ROSC) due to the presence of systemic ischemia-reperfusion [13]. It is considered that this systemic inflammatory change is associated with poor clinical prognosis after CA [14, 15]. Several studies have proposed that the novel inflammatory marker neutrophil-lymphocyte ratio (NLR) and the immature/total granulocyte (I/T-G) ratio is one of the predictors for the prognosis of CA [16–19]. Recently, gamma-glutamyl transpeptidase (GGT) to platelet ratio (GPR), which is easy to calculate and obtain, has been proposed as an inflammatory marker [20]. In cardiovascular disease, it has been found that the GPR can serve as an independent predictor of prognosis in coronary heart disease (CHD) patients undergoing percutaneous coronary intervention (PCI), and elevated GPR is associated with increased all-cause mortality and cardiovascular mortality [21]. A prospective study also found that higher GPR levels are associated with a higher risk of mortality in coronary artery disease (CAD) patients during a median follow-up period of 7.56 years [22]. Elevated GGT levels are considered a marker of inflammation and oxidative stress, which is increasingly recognized in cardiovascular diseases [23, 24]. A cohort study showed that GGT was positively associated with the risk of sudden cardiac death in the general male population during a mean follow-up of 22 years [25]. GGT is also associated with acute myocardial ischemia, which is the most common cause of fatal arrhythmia [26]. In systemic inflammation, the disruption of endothelial integrity promotes platelet adhesion and aggregation, resulting in the decrease of platelet count [27]. A reduction in platelet count was reported to be associated with a higher risk of mortality and unfavorable neurologic outcome at 6 months after CA [28]. Since GGT and platelets are involved in both inflammation and CA, we speculate that GPR may be associated with the prognosis after CA. However, so far, there is no relevant research report. Therefore, in the present study, we aimed to explore the associations between GPR and the outcomes after CA.

## 2. Materials and Methods

### 2.1. Study Population

In this study, the data we used were all obtained from the Dryad digital repository (10.5061/dryad.qv6fp83). Overall, this was a retrospective cohort study conducted from January 2007 to December 2015 at a single center (Erasme Hospital, Brussels, Belgium). The included cases were CA patients treated in the intensive care unit of this hospital. Because of its retrospective nature, there is no need for informed consent. Full information on the study population has been described in detail previously [29]. This study included comatose patients (Glasgow Coma Scale < 9) caused by IHCA or OHCA. Exclusion criteria include deaths within 24 hours of admission and lack of liver function data. All patients with CA and coma received a 24-hour target temperature management (TTM) aimed at a target temperature of 32-34°C. Midazolam and morphine were used as sedative drugs for deep sedation, and cisatracurium was used to control shivering. Repeated transesophageal and/or transthoracic echocardiography was used to assess cardiac function. Postresuscitation management was performed as previously described [30].

### 2.2. Data Collection

The following basic clinical characteristics data of the patient were collected in detail: demographic data, past chronic diseases, initial heart rhythm, bystander cardiopulmonary resuscitation (CPR), ROSC time, and total adrenaline application dose. After admission, the Acute Physiological and Chronic Health Assessment (APACHE) II score [31] and the Sequential Organ Failure Assessment (SOFA) score [32] were calculated according to the standards to evaluate the severity of the disease at the early stage of admission. The operating standards of laboratory examinations were carried out following local regulations, using the first blood sample taken after ROSC after admission. Laboratory testing indicators included the following: platelet (PLT), gamma-glutamyl transpeptidase (GGT), total bilirubin (TBIL), lactate dehydrogenase (LDH), alkaline phosphatase (ALP), aspartate (AST) and alanine (ALT) transaminases, prothrombin time (PT), fibrinogen, and international normalized ratio (INR). GPR was calculated as the ratio of gamma-glutamyl transpeptidase to platelet. Length of ICU stay, the use of vasoactive drugs, and mechanical ventilation were recorded. Comorbidities include hypertension (HTN), diabetes mellitus, chronic heart failure (CHF), chronic renal failure (CRF), CAD, neurological disease, liver cirrhosis, chronic obstructive pulmonary disease (COPD)/asthma, long-term use of corticosteroids, or anticoagulants. Record the use of drugs/interventions that may have hepatotoxicity, including quinolone, *β*-lactam, antiepileptic, isoniazid, pyrrole, trimethoprim/sulfamethoxazole, acetaminophen, amiodarone, and metronidazole.

### 2.3. Definitions

Hypertension was defined as systolic blood pressure ≥ 140 mmHg and diastolic blood pressure ≥ 90 mmHg (or currently being treated with antihypertensive medications) [33]. DM status was defined by several criteria: the previous history of diabetes mellitus or current use of hypoglycemic agents, fasting plasma glucose ≥ 7.0 mmol/L (≥126 mg/dL), or hemoglobin A1c ≥ 6.2% [34]. Acute liver failure was defined as encephalopathy of any degree with a prothrombin time prolongation of approximately 4-6 seconds or an international normalized ratio ≥ 1.5 [35]. Hypoxic hepatitis was defined as an elevation of AST and/or ALT to more than 20 times the upper limit of the normal range (≤50 IU/L) in the absence of other causes of hepatocellular necrosis [36]. Acute renal failure was diagnosed according to Acute Kidney Injury Network (AKIN) criteria with reference to serum creatinine levels [37]. The shock was defined as SBP ≤ 90 mmHg or the use of dopamine, norepinephrine, and epinephrine for more than 6 hours and the application of an intra-aortic balloon pump.

### 2.4. Ascertainment of Outcomes

The primary outcome of the study was all-cause mortality during ICU stay (ICU mortality). In-hospital mortality as well as the poor neurological functional outcome at 3 months after CA were defined as secondary endpoints. Assessment of neurological function was performed at 3 months after cardiac arrest by the cerebral performance category (CPC) score during follow-up (1, no or mild neurological dysfunction; 2, moderate neurological dysfunction; 3, severe neurological dysfunction; 4, vegetative state; and 5, death) [38]. Based on the scoring results, CPC 3-5 indicate a poor neurological outcome and CPC 1-2 are considered a good neurological outcome [39].

### 2.5. Statistical Analysis

Statistical analysis was performed using SPSS 26 (IBM SPSS, Armonk, New York, NY, USA), MedCalc 19.6.0 (MedCalc Software, Mariakerke, Belgium) and R 4.0.3 software (R Foundation for Statistical Computing, Vienna, Austria). A two-tailed *P* value of <0.05 was defined as statistically significant. For all continuous variables, the *Kolmogorov-Smirnov test* was used to assess whether they were normally distributed. Data with normal distribution were presented as means ± SD, and one-way analysis of variance was applied to determine statistical differences among groups. For those data that were not normally distributed, we expressed it using the median (lower quartile, upper quartile), and the comparison of differences among the groups was performed using the *Kruskal-Wallis test*. We present categorical variables as percentages, and comparisons were made using the *chi-square test* or the *Fisher exact test*. We established logistic regression models to assess the effect of GPR on three outcomes. We corrected for age, male gender, CHF, CRF, adrenaline, lowest ScvO_2_, lactate value at admission to ICU, bystander-witnessed CA, bystander CPR, ScvO_2_/SvO_2_ at admission, AST, ALT, LDH, and PAL as potential confounders. Receiver operating characteristic (ROC) curves were plotted. The predictive validity of GPR on outcomes was evaluated by the area under the curve (AUC). Likelihood ratio test (LRT) and the Akaike Information Criterion (AIC) were utilized for model comparison.

## 3. Results

### 3.1. Baseline Characteristics

Of the total 435 CA patients, there were 51 early deaths and another 30 patients who lacked the necessary information were excluded. Finally, 354 patients were included in the analysis. There were divided into three groups according to tertiles of calculated GPR values (low GPR group, <0.245, *n* = 119; middle GPR group, 0.245-0.486, *n* = 117; high GPR group, >0.486, *n* = 118). Baseline clinical characteristics of the present study population are shown in Table 1. There was no significant difference between the three groups in terms of demography and corticoids, except that the proportion of men in the middle GPR group was higher and in the high GPR group had more patients with liver cirrhosis and corticosteroid use (Table 1). During ICU stay, patients in the high GPR group had more frequently developed shock and acute liver failure, were more likely to receive vasopressin and inotropic drugs, and had a lower minimum platelet count(Table 1). For the laboratory findings on admission, there were significant differences among the three groups in AST, LDH, ALP, GGT, total bilirubin, PT, INR, and PLT (all *P* < 0.05) (Table 1). In Pearson correlation analysis, significant correlations were found between GPR and lactate (*r* = 0.203, *P* < 0.001), ALP (*r* = 0.381, *P* < 0.001), and total bilirubin (*r* = 0.161, *P* = 0.002) (Table 2).

### 3.2. Clinical Outcomes

**Figure 1:**
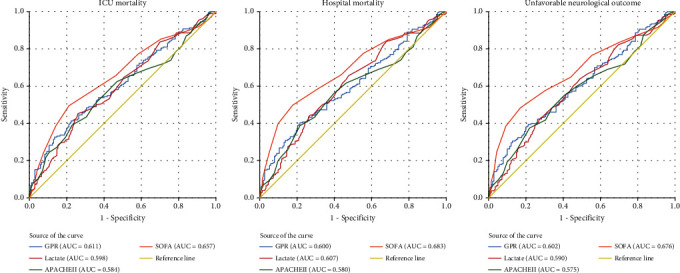
ROC curves for predicting ICU mortality, hospital mortality, and unfavorable neurologic outcome.

Of the 354 patients, 184 (52%) patients died during their ICU stay, of which there was higher ICU mortality in the high GPR group (65.3%). 201 (56.8%) patients died during hospitalization, with the highest rate of hospital mortality in the high GPR group (69.5%). 141 (39.8%) patients presented an unfavorable neurologic outcome, with the highest rate in the high GPR group (72.0%) (Table 3). We analyzed the relationship of GPR as a hierarchical variable with mortality and unfavorable neurologic outcomes, with the low GPR group as the reference. In univariate analysis, high GPR was all associated with the three outcomes (Table 4). This relationship remained significant in multivariate logistic regression analysis for the three outcomes (Table 4). In multivariate logistic regression analysis with GPR as a continuous variable, GPR was still associated with the three outcomes (Table 5). The ROC curve results showed that the AUC was 0.611 (95% Cl: 0.558–0.662) for ICU mortality, 0.600 (95% Cl: 0.547–0.651) for hospital mortality, and 0.602 (95% Cl: 0.549–0.653) for unfavorable neurologic outcomes (Figure 1).

### 3.3. Adding the GPR to Clinical Information

As shown in Table 6, we built two multivariate models and evaluated the models using likelihood ratio (LR) *χ*^2^ values, Akaike Information Criterion (AIC), and AUROC. For the three clinical outcomes, the likelihood ratio (LR) *χ*^2^ values for model 2 were all larger than those for model 1. For ICU mortality, the AIC values in models 1 and 2 were 441.471 and 427.015, respectively, and the AUROC increased from 0.734 in model 1 to 0.761 in model 2. For hospital mortality, the AIC values were 444.029 and 431.416 in model 1 and model 2, respectively, and the AUROC increased from 0.721 in model 1 to 0.749 in model 2. For the unfavorable neurologic outcome, the AIC values were 444.160 and 433.726 in model 1 and model 2, respectively, and the AUROC increased from 0.706 in model 1 to 0.733 in model 2. These all suggested that combining GPR with model 1 could be a better prognostic prediction model.

## 4. Discussion

In this study, we explored for the first time the relationship between GPR and prognosis after CA. Our results indicated that higher GPR was independently associated with higher ICU mortality and hospital mortality and unfavorable neurologic outcome after CA. And this relationship remained for GPR after adjustment for other confounders. GPR, as a new clinical marker, was able to increase the predictive ability of the original model for prognosis when it was added to the original model. Furthermore, as our study population included patients with IHCA and OHCA, this increases the scope to which the present findings apply.

In recent years, the role of hematological markers in cardiovascular diseases has been increasingly recognized. New inflammatory markers, such as the lactate/albumin ratio, I/T-G ratio, and NLR have been shown to be one of effective biomarkers for predicting the prognosis of CA patients [16–19, 40]. GPR as a new clinical biomarker was first proposed in 2016 to be one of the predictors of liver fibrosis and cirrhosis in patients with chronic hepatitis B virus infection. Subsequently, multiple studies have demonstrated the predictive value of GPR for the prognosis of patients with liver cancer and acute/chronic liver failure [41–44]. The results of a retrospective cohort study indicate that GPR is an independent predictor of adverse outcomes in patients with CHD after PCI [21].

The GPR is easily available and rapidly evaluated and can provide a reference for prognosis at an early stage after CA. In this study, we developed a multivariate model, which could better reflect the relevant information of poor prognosis after CA. GPR as a continuous variable could be considered as one of the independent predictors of ICU mortality (OR: 1.738, *P* = 0.002), hospital mortality (OR: 1.676, *P* = 0.005), and unfavorable neurologic outcome (OR: 1.623, *P* = 0.010). In addition, there was a significant increase when combining the GPR with the clinical information in the model (AUC values of 0.761, 0.749, and 0.733, respectively). This suggests that the addition of GPR to clinical information can indeed improve model performance. However, caution is still required for the direct application of GPR into clinical practice.

GGT is an important enzyme involved in the gamma-glutamyl cycle in amino acid absorption. The enzyme has a wide distribution in the body and, in addition to its presence in liver tissue, is also found in tissues such as the kidney, pancreas, and heart. It is elevated during decompensation from acute hepatitis, chronic active hepatitis, and cirrhosis. GGT plays a crucial role in the metabolism of glutathione, the most important cellular antioxidant in humans, and elevated levels of GGT are considered a marker of inflammation and oxidative stress [23, 24]. GGT has also been reported to be involved in ischemia-reperfusion injury [45]. Experimental results have shown that GGT levels rise in an isolated rat heart ischemia/reperfusion model. In the inflammatory process, GGT upregulates and increases antioxidant capacity and may cause leukotriene-induced inflammation [46]. The relationship between GGT and cardiovascular disease has been revealed in previous studies, and GGT has been confirmed to play a role in the occurrence and development of CVD [47]. In a meta-analysis including seven studies with a total of 273,141 participants, the results indicated that there was an association between GGT and the occurrence of cardiovascular disease and all-cause mortality [48]. In a prospective cohort study that enrolled 1780 men and followed for up to 22 years, GGT was positively associated with the future risk of sudden cardiac death in the general male population [25]. In our results, the high GGT level in the high GPR group was also associated with a worse prognosis. It is speculated that GGT plays a role in the pathophysiological mechanism after the onset of CA and has an impact on the clinical outcome of patients.

High GPR in our results also corresponded to low platelet counts as well as poor prognosis. In fact, platelet counts are reduced early after CA [28, 49, 50]. In patients after cardiac arrest and resuscitation, ischemia and hypoxia as well as increases in thrombin and catecholamines are considered activators of platelets [51]. Insufficient oxygen supply during and after cardiac arrest can lead to systemic inflammation and vascular endothelial dysfunction, resulting in enhanced vascular permeability and thrombosis [15]. Studies have shown that cardiac arrest causes blood-brain barrier damage, and systemic inflammatory response after cardiac arrest may aggravate cerebral ischemic injury [52]. Gando and Wada found that ischemia-reperfusion injury and successful resuscitation caused by CA were associated with endothelial cell activation and were associated with the prognosis of patients with OCHA [51]. Bro-Jeppesen et al. reported that endothelial damage and activation were found within the first 72 h after CA and that endothelial damage was associated with a high baseline systemic inflammatory level [53]. Systemic ischemia-reperfusion injury after CA causes systemic inflammatory response syndrome (SIRS) [35]. Disseminated intravascular coagulation (DIC) is a common complication of SIRS, which occurs after cardiac arrest and resuscitation [54]. Massive activation of the coagulation pathway along with massive consumption of platelets is the most prominent pathological feature of DIC [55]. Therefore, DIC can lead to extensive microvascular embolization after resuscitation from cardiac arrest [14]. The findings of Kim et al. suggest that an early increased DIC score in OHCA patients is an independent predictor of early mortality and poor long-term prognosis [56]. Prolonged systemic ischemia-reperfusion may lead to cerebral and cardiac insufficiency, inadequate tissue oxygen supply, and immune and coagulation pathway activation and thus increase the risk of multiple organ failure [57]. Based on the above findings, the relationship between GPR and the prognosis of CA may involve various reasons. GPR is an indicator of the systemic inflammatory response; a reasonable explanation may involve the mechanism of inflammation after CA. However, the exact mechanism and role of GPR need further demonstration.

## 5. Limitations

Our study also has some limitations. First, only the test results of the first blood sample after admission were obtained from the database, without the test results of a certain time interval. Therefore, we cannot judge their causal relationship with clinical outcomes and can only establish statistical links. Second, as a single-center retrospective study, the generalizable application of the findings will be limited. Third, due to the limitation of the research design, some biases are unavoidable, and unknown confounders have the potential to influence the results despite adjusting for multiple confounders.

## 6. Conclusion

Elevated GPR on admission was significantly associated with ICU mortality, hospital mortality, and unfavorable neurologic outcome after CA. The predictive value of GPR after CA needs to be confirmed by further multicenter studies.

## Figures and Tables

**Table 1:** Baseline clinical characteristics.

Variable	Low GPR group (*n* = 119)	Middle GPR group (*n* = 117)	High GPR group (*n* = 118)	*P*
Mean age (years)	62 (52, 74)	62 (52, 73)	60 (51,75)	0.957
Gender (men), *n* (%)	79 (66.4)	95 (81.2)	80 (67.8)	0.021
Weight (kg)	78 (67, 85)	79 (70, 88)	75 (65,85)	0.279
*Arrest characteristics*				
Bystander-witnessed CA, *n* (%)	102 (85.7)	99 (84.6)	101 (85.6)	0.967
Bystander CPR, *n* (%)	75 (63.0)	83 (70.9)	81 (68.6)	0.409
Adrenaline, *n* (%)	103 (86.6)	107 (91.5)	107 (90.7)	0.416
Out of hospital, *n* (%)	74 (62.2)	64 (54.7)	63 (53.8)	0.362
TTM, *n* (%)	108 (90.8)	105 (89.7)	102 (86.4)	0.541
Noncardiac cause, *n* (%)	46 (38.7)	43 (36.8)	52 (44.1)	0.493
Nonshockable rhythm, *n* (%)	64 (53.8)	70 (59.8)	75 (63.6)	0.303
*Corticoids*				
Chronic heart failure, *n* (%)	23 (19.3)	25 (21.4)	29 (24.6)	0.614
Hypertension, *n* (%)	54 (45.4)	47 (40.2)	51 (43.2)	0.719
Coronary artery disease, *n* (%)	52 (43.7)	39 (33.3)	48 (40.7)	0.246
Diabetes, *n* (%)	27 (22.7)	35 (29.9)	23 (19.5)	0.160
COPD/asthma, *n* (%)	21 (17.6)	13 (11.1)	25 (21.2)	0.110
Neurological disease, *n* (%)	15 (12.6)	16 (13.7)	20 (16.9)	0.612
Chronic renal failure, *n* (%)	18 (15.1)	22 (18.8)	20 (16.9)	0.753
Liver cirrhosis, *n* (%)	1 (0.8)	2 (1.7)	9 (7.6)	0.007
Corticosteroids, *n* (%)	17 (14.3)	25 (21.4)	36 (30.5)	0.010
Chronic anticoagulation, *n* (%)	18 (15.1)	16 (13.7)	27 (22.9)	0.132
*During ICU stay*				
IABP, *n* (%)	7 (5.9)	6 (5.1)	11 (9.3)	0.394
ECMO, *n* (%)	11 (9.2)	18 (15.4)	16 (13.6)	0.347
Shock, *n* (%)	50 (42.0)	60 (51.3)	80 (67.8)	<0.001
Vasopressor therapy, *n* (%)	80 (67.2)	85 (72.6)	103 (87.3)	0.001
Inotropic agents, *n* (%)	51 (42.9)	64 (54.7)	78 (66.1)	0.002
Mechanical ventilation, *n* (%)	117 (98.3)	116 (99.1)	116 (98.3)	0.823
CRRT, *n* (%)	15 (12.6)	17 (14.5)	25 (21.2)	0.170
Paracetamol, *n* (%)	71 (59.7)	64 (54.7)	55 (46.6)	0.127
Amiodarone, *n* (%)	58 (48.7)	60 (51.3)	57 (48.3)	0.886
*β*-Lactams, *n* (%)	49 (41.2)	46 (39.3)	52 (44.1)	0.758
Quinolones, *n* (%)	3 (2.5)	3 (2.6)	0 (0.0)	0.217
Azoles, *n* (%)	2 (1.7)	3 (2.6)	3 (2.5)	0.873
Isoniazid, *n* (%)	—	—	—	NA
TMP/SMX, *n* (%)	—	—	—	NA
Metronidazole, *n* (%)	2 (1.7)	—	—	0.137
Chemotherapy, *n* (%)	—	—	—	NA
AKI, *n* (%)	63 (52.9)	72 (61.5)	75 (63.6)	0.210
HH, *n* (%)	6 (5.0)	8 (6.8)	12 (10.2)	0.308
ALF, *n* (%)	54 (45.4)	66 (56.4)	77 (65.3)	0.009
ICU length of stay (days)	5 (3, 8)	4 (2, 11)	4 (2, 9)	0.852
APACHE II score	25 (21, 29)	23 (19, 28)	24 (19, 30)	0.218
SOFA score	11 (9, 14)	11 (9, 13)	11 (9, 14)	0.499
Lowest ScvO_2_/SvO_2_ (%)	62.3 (57.7, 66.0)	62.0 (56.8, 67.0)	62.7 (54.0, 67.0)	0.948
Lowest platelet count (mm^3^)	166 (129, 216)	138 (93, 192)	89 (56, 129)	<0.001
*Laboratory findings on admission*				
Lactate (m Eql^−1^)	4.8 (3.8, 7.3)	5.10 (4.3, 7.4)	5.3 (4.1, 8.0)	0.319
CRP (mg dL^−1^)	31 (10, 66)	40 (14, 90)	44 (18, 98)	0.200
Creatinine (mg dL^−1^)	1.2 (0.9, 1.6)	1.3 (1.0, 1.6)	1.2 (0.9, 1.6)	0.595
ScvO_2_/SvO_2_ (%)	70.02 ± (8.90)	69.00 ± (8.60)	69.13 ± (10.31)	0.657
AST (IU/L)	65 (36, 169)	100 (51, 186)	115 (70, 239)	0.001
ALT (IU/L)	55 (28, 131)	72 (32, 142)	78 (39, 170)	0.062
LDH (IU/L)	295 (216, 425)	352 (249, 524)	372 (253, 534)	0.010
ALP (IU/L)	66 (52, 88)	70 (60, 102)	89 (64, 128)	<0.001
GGT (IU/L)	37 (22, 55)	73 (55, 88)	115 (82, 196)	<0.001
Total bilirubin (mg dL^−1^)	0.43 (0.29, 0.64)	0.50 (0.36, 0.85)	0.61 (0.39, 1.20)	<0.001
APTT (sec)	31.8 (26.1, 41.3)	32.1 (27.7, 46.0)	34.70 (28.4, 45.4)	0.168
PT (%)	69.28 ± (21.74)	59.25 ± (22.39)	63.31 ± (23.02)	0.002
INR	1.20 (1.08, 1.42)	1.27 (1.13, 1.53)	1.35 (1.20, 1.70)	0.001
Platelets (mm^3^)	238 (188, 322)	211 (158, 249)	137 (84.25, 190)	<0.001
Proteins (mg dL^−1^)	5.6 (5.1, 6.5)	5.8 (5.0, 6.3)	5.7 (5.0, 6.2)	0.872
Glucose (mg dL^−1^)	211 (169, 301)	210 (150, 297)	190 (147, 264)	0.132
pH	7.29 (7.22, 7.38)	7.31 (7.23, 7.38)	7.29 (7.19, 7.36)	0.552
PaCO_2_ (mmHg)	123 (86, 186)	111 (91, 162)	109 (83, 177)	0.510
PaO_2_ (mmHg)	38 (33, 45)	37 (32, 43)	37 (32, 43)	0.632
MAP (mmHg)	91 (76, 108)	90 (77, 104)	84 (75, 99)	0.093

Abbreviation: ICU: intensive care unit; CA: cardiac Arrest; CPR: cardiopulmonary resuscitation; ROSC: return of spontaneous circulation; TTM: targeted temperature management; COPD: chronic obstructive pulmonary disease; IABP: intra-aortic balloon pump; ECMO: extracorporeal membrane oxygenation; CRRT: continuous renal replacement therapy; HH: hypoxic hepatitis; ALF: acute liver failure; AKI: acute kidney injury; ScvO_2_/SvO_2_: central venous/mixed venous oxygen saturation; AST: aspartate aminotransferase; ALT: alanine aminotransferase; LDH: lactate dehydrogenase; ALP: alkaline phosphatase; GGT: *γ*-glutamyl transferase; APTT: activated partial thromboplastin time; PT: prothrombin time; INR: international normalized ratio; MAP: mean arterial pressure; CRP: C-reactive protein; APACHE: Acute Physiology and Chronic Health Evaluation; SOFA: Sequential Organ Failure Assessment.

**Table 2:** Correlation analysis.

Variable	*r* value	*P* value
Lowest ScvO_2_/SvO_2_ (%)	-0.046	0.387
Lowest platelet count (mm^3^)	-0.226	<0.001
Lactate (mEql^−1^)	0.203	<0.001
CRP (mg dL^−1^)	0.075	0.157
Creatinine (mg dL^−1^)	0.010	0.854
Glucose (mg dL^−1^)	-0.055	0.301
ScvO_2_/SvO_2_ (%)	-0.039	0.469
AST (IU/L)	0.104	0.050
ALT (IU/L)	0.093	0.081
LDH (IU/L)	0.093	0.082
ALP (IU/L)	0.381	<0.001
Total bilirubin (mg dL^−1^)	0.161	0.002
APTT	<0.001	0.997
PT (%)	-0.017	0.757
INR	0.033	0.541

Abbreviation: ALP: alkaline phosphatase; ALT: alanine aminotransferase; APTT: activated partial thromboplastin time; AST: aspartate aminotransferase; CRP: C-reactive protein; GGT: *γ*-glutamyl transferase; INR: international normalized ratio; LDH: lactate dehydrogenase; PLT: platelets; PT: prothrombin time; ScvO_2_/SvO_2_: central venous/mixed venous oxygen saturation.

**Table 3:** Primary and secondary outcomes.

Outcome	All patients (*n* = 354)	Low GPR group (*n* = 119)	Middle GPR group (*n* = 117)	High GPR group (*n* = 118)	*P*
ICU mortality, *n* (%)	184 (52.0)	51 (42.9)	56 (47.9)	77 (65.3)	<0.001
Hospital mortality, *n* (%)	201 (56.8)	59 (49.6)	60 (51.3)	82 (69.5)	0.003
Unfavorable neurologic outcome, *n* (%)	213 (60.2)	62 (52.1)	66 (56.4)	85 (72.0)	0.004

Abbreviation: ICU: intensive care unit.

**Table 4:** Logistic regression analysis for the GPR as a hierarchical variable.

Outcome	Baseline GPR	Unadjusted OR (95% CI)	*P*	Adjusted OR (95% CI)	*P*
ICU mortality	Low	Ref.		Ref.	
	Middle	1.224 (0.733, 2.045)	0.440	1.149 (0.644, 2.050)	0.639
	High	2.504 (1.482, 4.232)	0.001	2.162 (1.175, 3.981)	0.013
Hospital mortality	Low	Ref.		Ref.	
	Middle	1.070 (0.643, 1.783)	0.794	0.952 (0.537, 1.689)	0.868
	High	2.316 (1.361, 3.942)	0.002	1.915 (1.041, 3.524)	0.037
Unfavorable neurologic outcome	Low	Ref.		Ref.	
	Middle	1.190 (0.713, 1.987)	0.507	1.061 (0.601, 1.875)	0.838
	High	2.368 (1.381, 4.061)	0.002	1.954 (1.058, 3.610)	0.032

Abbreviation: CI: confidence interval; ICU: intensive care unit; OR: odds ratio.

**Table 5:** Logistic regression analysis for the GPR as a continuous variable.

Outcome	Unadjusted OR (95% CI)	*P*	Adjusted OR (95% CI)	*P*
ICU mortality	1.694 (1.225, 2.342)	0.001	1.738 (1.221, 2.474)	0.002
Hospital mortality	1.689 (1.199, 2.377)	0.003	1.676 (1.164, 2.413)	0.005
Unfavorable neurologic outcome	1.654 (1.166, 2.345)	0.005	1.623 (1.121, 2.351)	0.010

Abbreviation: CI: confidence interval; ICU: intensive care unit; OR: odds ratio.

**Table 6:** Comparison of different prognostic models on CA patients.

Model predictors	Likelihood ratio test *χ*^2^	AIC	*P*	AUROC
ICU mortality				
Model 1	64.724	441.471	<0.001	0.734
Model 2	81.179	427.015	<0.001	0.761
Hospital mortality				
Model 1	56.191	444.029	<0.001	0.721
Model 2	70.803	431.416	<0.001	0.749
Unfavorable neurologic outcome				
Model 1	47.842	444.160	<0.001	0.706
Model 2	60.275	433.726	<0.001	0.733

Abbreviation: AIC: Akaike Information Criterion; AUROC: area under the receiver operating characteristics curve; ICU: intensive care unit. Model 1: age, male gender, witnessed arrest, bystander CPR, adrenaline, lactate, LDH, lowest ScvO_2_. Model 2: add GPR to model 1.

## Data Availability

All relevant data is available via Dryad: 10.5061/dryad.qv6fp83
